# MYC, Cell Competition, and Cell Death in Cancer: The Inseparable Triad

**DOI:** 10.3390/genes8040120

**Published:** 2017-04-17

**Authors:** Simone Di Giacomo, Manuela Sollazzo, Simona Paglia, Daniela Grifoni

**Affiliations:** Department of Pharmacy and Biotechnology, University of Bologna, Via Selmi 3, 40126 Bologna, Italy; simone.digiacomo2@unibo.it (S.D.G.); manuela.sollazzo2@unibo.it (M.S.); simonapaglia.89@gmail.com (S.P.)

**Keywords:** MYC, cell competition, cancer, apoptosis, *Drosophila*

## Abstract

Deregulation of MYC family proteins in cancer is associated with a global reprogramming of gene expression, ultimately promoting glycolytic pathways, cell growth, and proliferation. It is well known that MYC upregulation triggers cell-autonomous apoptosis in normal tissues, while frankly malignant cells develop resistance to apoptotic stimuli, partly resulting from MYC addiction. As well as inducing cell-autonomous apoptosis, MYC upregulation is able to trigger non cell-autonomous apoptotic death through an evolutionarily conserved mechanism known as “cell competition”. With regard to this intimate and dual relationship between MYC and cell death, recent evidence obtained in *Drosophila* models of cancer has revealed that, in early tumourigenesis, MYC upregulation guides the clonal expansion of mutant cells, while the surrounding tissue undergoes non-cell autonomous death. Apoptosis inhibition in this context was shown to restrain tumour growth and to restore a wild-type phenotype. This suggests that cell-autonomous and non cell-autonomous apoptosis dependent on MYC upregulation may shape tumour growth in different ways, soliciting the need to reconsider the role of cell death in cancer in the light of this new level of complexity. Here we review recent literature about MYC and cell competition obtained in *Drosophila*, with a particular emphasis on the relevance of cell death to cell competition and, more generally, to cancer. Possible implications of these findings for the understanding of mammalian cancers are also discussed.

## 1. Introduction

Organs are composed of several cell typologies that experience complex relationships and modify their behaviour to survive in the changing context. Cancer itself can be seen as an evolving landscape, in which tumour cells may be either hampered or supported by different neighbours in the disruption of tissue homeostasis. Understanding the molecular basis of the social cell biology of cancer is thus indispensable to develop novel therapeutic approaches targeting the whole cancer community. The oncoprotein MYC plays instrumental roles in development and cancer, strictly dependent on its ability to promote both cell growth and cell death in different genetic backgrounds. In recent years, these two intrinsic features of the MYC protein were shown to merge in a unique trait named “cell competition”, an evolutionarily conserved mechanism ultimately governing cell selection in organ development and, possibly, in cancer.

## 2. MYC and Cell Growth 

The oncoprotein c-MYC is known to regulate a number of cellular processes, from cell growth to apoptosis and metabolism [1]. The *Drosophila* protein, namely dMYC (hereafter referred to as MYC), shows poor sequence similarity with its human counterpart [2], but *Drosophila* MYC and vertebrate c-MYC can, however, substitute each other in several experimental systems, thus demonstrating functional conservation [3,4]. As in mammals, most MYC transcriptional activity is carried out through dimerisation with its binding partner Max [5]; the MYC/Max/Mad network consists of single MYC, Max, and Mad/Mxd components in the fly [6], making it a simple system for investigating the complexity of MYC function. 

MYC is encoded by the *diminutive* (*dm*) *locus* [2], and its over-expression in discrete territories of the *Drosophila* larval imaginal wing disc epithelium, the primordium of the adult wing and thorax [7], induces mass accumulation by accelerating the G1/S transition of the cell cycle [8]. By contrast, hyper-proliferation is prevented, as entry into the M-phase is limited by the availability of the phosphatase String/CDC25. On the other hand, *dm* hypomorphic mutants show developmental delay and small body size [8], while null mutants barely complete embryo development [9]. MYC’s role in cell growth is largely carried out through the modulation of ribosome biogenesis [10], and it also regulates organismal growth downstream of the Insulin/TOR signalling pathway [11,12,13] and of the ecdysone signalling [14]. MYC has also been involved in tissue regeneration through the Wg/Wnt pathway [15], and its transcriptional activation is modulated, among others [16], by the Hippo (Hpo) signalling pathway [17,18], known to regulate tissue and organ growth from *Drosophila* to mammals [19]. The downstream effector of the Hpo pathway is Yorkie (Yki), encoded by the *Drosophila* homologue of the human *YAP* (Yes-Associated-Protein) oncogene, which, following deregulation of upstream components of the pathway, activates, besides MYC, transcription of several genes involved in cell growth, proliferation, and survival such as *cyc E*, *dIAP1* (*Drosophila Inhibitor of APoptosis 1*), and the miRNA *bantam* [20]. Finally, another essential aspect impacting MYC function is protein stability: in *Drosophila*, as well as in mammals, MYC protein shows a half-life of about 30 min, and several enzymatic activities have been found to modulate its biological activity by targeting different residues within its degron [21,22,23,24,25].

## 3. MYC and Cell Death

A moderate increase in MYC levels can stimulate cellular and organismal growth, whereas excessive MYC activity is able to induce cell-autonomous apoptosis, with different thresholds in different physiological conditions and genetic backgrounds [26,27]. This double face of MYC has fostered a series of studies aimed at unravelling a seeming paradox (reviewed in [28]). In vertebrates, several members of the anti-apoptotic BCL family have been found to mediate MYC-driven apoptosis, either in lymphoid malignancies [29,30,31] or in solid tumours [32]. BCL family proteins are in turn regulated by p53, a manifold modulator of apoptosis in higher organisms [33]. p53 has been found to mediate MYC-induced apoptosis in murine models of lymphomagenesis [34,35] and in neuroblastoma [36], but has also shown to be dispensable for MYC-mediated cell death in several different contexts [37,38,39]. MYC’s ability to trigger apoptosis is also conserved in *Drosophila*. Different c-MYC isoforms differently regulate cell growth and apoptosis in *Drosophila* [3], and induction of high levels of MYC in the imaginal eye and wing epithelia triggers massive cell death [40,41]. Consistently, reduction of MYC levels protects these cells from apoptotic death following irradiation [41,42]. Despite a significant increase of *p53* transcript upon MYC induction, *Drosophila* p53 has been proven to be partly dispensable for MYC-dependent, cell-autonomous apoptotic death, which rather involves the RHG group’s effectors: Reaper (Rpr), Head involution defective (Hid) and Grim, whose expression may be directly induced by MYC [41]. These proteins carry out their pro-apoptotic function by negatively regulating the *Drosophila* pro-survival effector dIAP1 [43] which, in turn, inhibits the Caspase 9-like Dronc [44]. However, an intergenic region in the RHG block, containing a p53 responsive element [45], has been found to mediate MYC-driven apoptosis; animals lacking this region indeed undergo extensive overproliferation upon MYC overexpression [46]. In addition, the Hpo pathway has been shown to downregulate the pro-apoptotic gene *rpr* through Yki and p53, although the role of MYC has not been investigated in this study [47]. 

## 4. MYC Enters Cell Competition

Cell competition (CC) is described as a mechanism of cell fitness comparison aimed at eliminating suboptimal cells, supporting tissue homeostasis. CC was first reported by Morata and Ripoll in the 1970s while studying the growth rates of cells bearing ribosomal gene mutations (*Minute*, *M*) in the *Drosophila* wing disc [48,49], a larval organ consisting in a monolayer of highly proliferating epithelial cells [7]. *Minute* heterozygous flies (*M*/+) display a normal morphology and are viable and fertile, although their organs show low proliferation rates compared to wild-type flies, resulting in developmental delay. Using genetic mosaic techniques [50], the authors generated wild-type clones in *M*/*+* flies and observed that *M*/*+* cells were eliminated by surrounding wild-type cells, which sometimes composed the entire adult organ [48,51]. CC was then proposed as a mechanism of surveillance through which viable, but sub-optimal cells (*losers*), could be detected and out-competed by fitter neighbours (*winners*), mainly through c-Jun N-terminal kinase (JNK)-mediated apoptosis [51,52]. CC was observed for the first time in mice in 2004, when Oliver and colleagues found that the *Belly spot and tail* (*Bst*) phenotype was due to a mutation in a gene encoding a ribosomal protein: *Bst* mutant cells were eliminated by wild-type cells during the development of chimeric blastocysts [53], recapitulating the phenomenon observed in *Drosophila*. 

**Figure 1:**
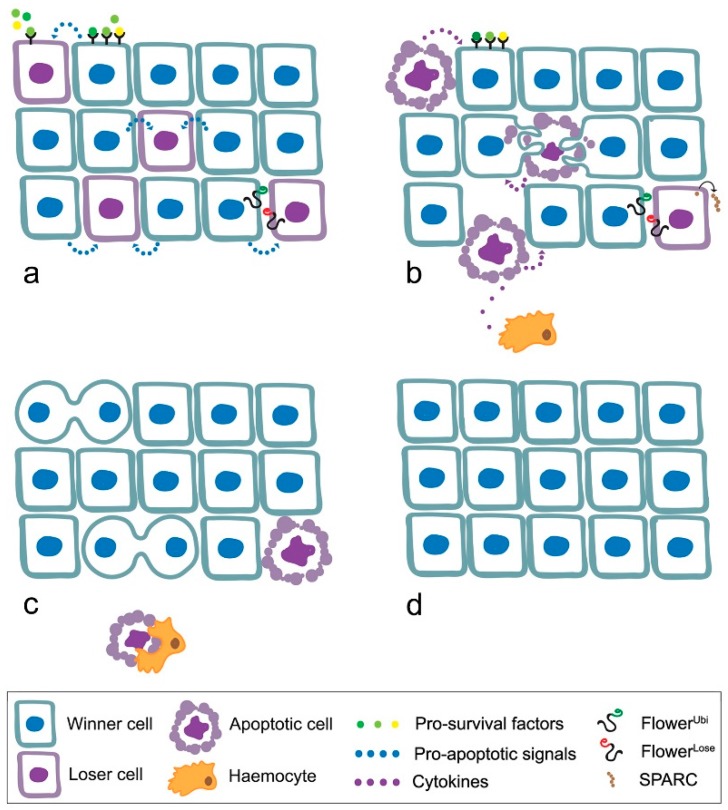
Mechanisms of cell competition in developing organs. In developing organs, cell competition participates in the maintenance of tissue homeostasis. Loser cells show insufficient capability to capture morphogens and growth factors (**a**), display specific molecular signatures (**b**) and are engulfed by adjacent cells (**c**) or extruded from the tissue and eliminated by recruited haemocytes; (**d**) At the end of development, the tissue results composed of cells showing comparable fitness, as winner cells overproliferate as to fill the space left by the losers.

Since then, the scientific community has been working to decipher the intricate relationships intervening between winner and loser cells, leading to the identification of a number of traits, central to CC (Figure 1), summarised in the following in-progress list:

- competitive interactions are established when mutational events occur in a cell that reduce or increase its fitness in the context [8,54];
- loser cells suffer from shortage of survival/growth factors such as the *Drosophila* TGFβ orthologue Decapentaplegic (Dpp) [52];
- cells engaged in the competitive event release soluble factors [55] and express specific genetic fingerprints that confer them a loser or winner state [56,57,58];
- loser cells undergo JNK-dependent apoptosis due to the low levels of survival signals and to the expression of the pro-apoptotic gene *hid* [40,52];
- the high contact tension at the interface of winner and loser cell leads to the elimination of loser cells through cell-cell intercalation [59];
- local tissue crowding can induce mechanical competition, independent of known markers of cell fitness [60];
- winner cells can acquire the ability to engulf adjacent losers [61];
- the most part of the loser cells is extruded from the tissue and recruits professional haemocytes, responsible for the elimination of cell debris [62,63];
- elimination of the loser cells leads to overproliferation of the winners [54].

As described in Chapter 2, MYC protein plays a central role in defining cell size and organ growth. Studies in *Drosophila* have highlighted that cells with low MYC levels display a growth detriment; on the other hand, high MYC levels are sufficient to promote cell growth [8,9]. In 2004, two studies first described the competitive properties of high MYC-expressing cells [40,54]. In the wing disc epithelium, cells expressing high MYC levels were able to induce apoptotic death of the wild-type neighbours and to overgrow as to fill the space left by the losers, hence the concept of super-competition [40,54]. It has also been shown that loser and winner cells do not need to physically interact to confront their relative fitness; still uncharacterised soluble factors are indeed produced following co-culture of cells displaying different MYC levels that are sufficient to induce competitive behaviours in the confronting cells [55]. An in silico study has identified 9 miRNAs involved in CC that mainly target elements of the JNK pathway, suggesting that winner and loser cells may exchange molecular information through the release of exosomal vesicles [64]. In 2009, Rhiner and colleagues found a physiological role for MYC-mediated cell competition in guiding differentiation of the germline stem cells (GSCs) in the *Drosophila* ovary. In this case, the loser GSCs were not eliminated by apoptosis, but were committed to leave the niche and to differentiate. This mechanism was driven by MYC-mediated Dpp signalling [65]. It is also known that MYC over-expressing cells need a wild-type p53 function to acquire a winner fate: it was indeed found that loss of p53 impairs their metabolism and reduces viability, thus preventing CC [66].

A cell’s fortune in CC seems thus dictated by a multitude of signals, among which Flower (Fwe), a trans-membrane protein, has been shown to play a role by labelling winner and loser cells with different variants: Fwe^Ubi^ is constitutively expressed throughout the disc epithelium and downregulated in dying cells, which express Fwe^Lose^ isoforms instead [56]. Fwe^Lose^ expression seems essential to establish cell death, as knockdown of the Fwe^Lose^ isoforms rescues the loser phenotype [56]. Secreted Protein Acidic and Rich in Cysteine (SPARC), a matricellular glycoprotein upregulated in loser cells in the early steps of CC, was rather demonstrated to exert a transient self-protective effect by increasing the threshold for caspase activation [57]. Another determinant of cell fate in CC is *ahuizotl* (*azot*), a gene whose transcription integrates Fwe^Lose^ and SPARC information, monitoring loser cell elimination. Therefore, *azot* acts as a checkpoint of cell fitness marking cells that will be eliminated, and its activity is restricted to competitive phenomena in several organs [58]. 

It has recently emerged that MYC-mediated CC is conserved in mammalian development. It has indeed been shown that mouse epiblast is naturally composed of cells with different MYC expression: the embryonic stem cells with higher MYC levels overgrow and out-compete cells with lower MYC activity, which undergo apoptotic death [67,68]. Evidence of MYC-mediated CC was also found in the mammalian heart: CC acts by expanding MYC over-expressing cardiomyocytes while replacing the wild-type neighbours. The competitive replacement is phenotypically silent, as it does not hinder normal heart functions [69,70]. Studies on CC in mammals, although at an early stage, seem thus to recapitulate what happens in *Drosophila* models of MYC-mediated CC. For this reason, further investigation of this phenomenon in the fruitfly may give relevant information on the molecular mechanisms at the basis of tissue regeneration and cancer.

## 5. MYC, Cell Competition and Cancer

The nature of CC entails the active participation of different cell populations, struggling for resources and space while growing in close proximity within a tissue [71]. This is also a key trait of cancer, where clonal growth is promoted as a response to active selection [72,73]. MYC activation is considered a hallmark of cancer initiation and maintenance [74], and the discovery of MYC function in CC has primed a series of speculations about a possible role for this phenomenon in cancer [71,75,76,77,78]. Tumours undergo continuous genetic diversification and epigenetic plasticity followed by clonal selection and expansion, revealing a genetic architecture reminiscent of Darwin’s evolutionary trees [79]. Given its double function in cell elimination and replacement, MYC-mediated CC may thus represent one of the forces driving both clonal culling and dominance during cancer progression. Our previous studies indeed showed that, in *Drosophila* epithelia, cells bearing mutations affecting epithelial cell polarity display low levels of MYC and are eliminated from the tissue through JNK-mediated apoptosis [80]. In line with this evidence, a recent study has demonstrated that low levels of MYC fail to upregulate the JNK repressor *puckered* (*puc*), thus promoting JNK-mediated cell death [81]. In addition, MYC overexpression was sufficient to rescue those cells from competitive death and to turn them into super-competitors, able to grow while killing adjacent wild-type cells [80]. As described above, MYC activity is regulated, among others, by the Hpo pathway [17,18]. Its function in restraining cell and tissue growth is responsible for the out-competition of polarity-deficient cells in *Drosophila*, given that Yki overexpression provides these cells with the capability to escape untimely elimination and to form tumours [82,83,84]. This evidence has finally explained how mutations of genes encoding polarity proteins can lead to unrestrained proliferation [85]. The Hpo pathway was also shown to participate in the oncogenic cooperation between activated Ras and loss of cell polarity in *Drosophila* cancer models [82,83,86]. In this case, Yki nuclear accumulation was visible in the cancer tissue together with an upregulation of its target genes, including *MYC* [82]. A very recent study in *Drosophila* further demonstrated that CC drives neoplastic transformation in an EGFR-mir8 cooperative model through the formation of MYC-overexpressing giant cells [87]. In addition, two studies have underlined the importance of the relative cell numbers in priming CC: potential winners can be eliminated when sporadic in the tissue [82] and, on the other hand, if a substantial number of potential losers are found in a field, they can grow and overwhelm the opposing cells [88]. These complex dynamics go well beyond the genetic structure of the participating cells, and it is conceivable that new findings will come from the analysis of MYC-mediated CC between different cell species, such as cancer and stromal cells composing the tumour microenvironment.

## 6. Apoptotic Cell Death in Cancer: What Side Does It Stand on?

**Figure 2:**
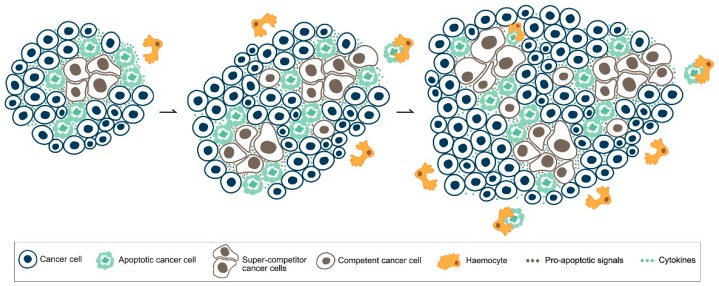
Hypothetical mechanisms of intra-tumoural cell competition. A cancer sub-field is represented in which super-competitor cells (brown), such as those upregulating MYC, form competitive niches that induce apoptotic death in the surrounding weaker cells (green). As cancer grows, new competitive niches develop from cells competent to exploit the signals coming from dying cells. Elimination of the loser cells gets to be inefficient, given their exponential increase in number within the cancer mass.

Evasion of apoptosis is a hallmark feature of tumour cells [89], and reactivation of cell death programmes is a common strategy in cancer treatment [90]. The traditional concept of apoptosis is based on a mechanism through which cell dictates its own demise in an autonomous manner, but emerging findings in several experimental models open up to a variety of non-autonomous regulations of apoptosis that may play counterintuitive roles in cancer [91]. As an example, it is known that compensatory proliferation occurs following apoptotic death in *Drosophila* [92,93], where activation of the initiator Caspase 9-like Dronc, beside inducing cell destruction, is known to stimulate the JNK signaling, that is required for cancer growth [92,94,95]. The same phenomenon has been observed in inflammation-induced fly models of tumourigenesis [96] and in mice cancer models, where dying neoplastic cells contributed to tumour repopulation following radiation or chemotherapy by secreting the growth factor PGE_2_ [97,98]. CC, as described in Chapter 4, is one of the mechanisms that trigger apoptosis in a non-autonomous manner, and cell death is necessary for the winner cells to repopulate the field in a developmental context [54,61]. Focusing on the vision that cancers behave as deranged organs [99], it is plausible that signals emanating from the out-competed cells are intercepted and exploited by cancer cells to enhance their performance, thus hijacking cell death to their own benefit. An example of this behaviour can be found in a recent study by Suijkerbuijk and colleagues, where the MYC-dependent growth of intestinal adenomas due to CC was hampered by apoptosis inhibition [100]. This evidence discloses a role for non-autonomous apoptosis in facilitating the growth of pre-neoplastic masses, suggesting that apoptosis inhibitors should be explored as possible therapeutic agents to contain cancer mass and prevent organ failure. Our lab is currently studying the consequences of intra-tumoural MYC-mediated CC in *Drosophila*, and our data reveal that this phenomenon is able to shape the final cancer mass in an apoptosis-dependent manner [101]. Our findings suggest that CC is an innate process governing both cancer initiation and progression, where cell death fuels the clonal expansion of the fittest cells in the context. CC and apoptosis thus appear to be strictly linked one another, and emerge as fundamental cancer drivers also in a computational model of tumour growth, where several parameters of malignancy such as intra-tumour heterogeneity and accelerated repopulation have been taken into account [102]. In mammals, the oncogenic properties of the apoptotic cells have been successfully investigated in a mouse model of B-cell lymphoma, where dying cells have been found to promote angiogenesis and to process the extracellular matrix, further to fuel tumour growth [103]. A number of studies correlated the apoptotic index (AI) to a poor prognosis in several types of cancer [104,105,106]. While authors find an explanation in that tumours with a high AI need more time to reach a relevant mass, thus accumulating further detrimental mutations, we suggest that apoptotic cells, when massively present, stall the engulfment machinery and persist in the tissue, where they contribute to tumour growth by secreting pro-mitogenic molecules (Figure 2). In this direction, dying glioma cells were recently found to promote angiogenesis through a Caspase 3-dependent VEGF regulation, so favouring cancer recurrence [107]. Induction of MYC-mediated CC in different experimental models and genetic backgrounds may thus represent an invaluable tool to characterise the local and systemic consequences of apoptotic cell death in cancer development, from initiation to unrestrained growth and metastasis.

## 7. Final Remarks

Cells cooperate to build an organ and, in a similar way, they cooperate to build a cancer. Although the contexts are impressively distant, MYC-mediated cell competition seems to be at work in both cases with the same basic, sequential elements: cell–cell disparity in MYC contents, death of the cells with lower MYC levels, and proliferation of the cells with higher MYC levels. This stereotypical module shapes organ development and, possibly, cancer evolution. In growing tumours, an excess of dying cells is known to contribute to mass expansion, but the implication of MYC-mediated cell competition in this cancer trait has just begun to be investigated. Further research is warranted on the intricate “life and death” signals exchanged by confronting cell populations within the cancer community.
